# Network Theoretical Approach to Explore Factors Affecting Signal Propagation and Stability in Dementia’s Protein-Protein Interaction Network

**DOI:** 10.3390/biom12030451

**Published:** 2022-03-15

**Authors:** Amit Kumar Lalwani, Kushagra Krishnan, Sali Abubaker Bagabir, Mustfa F. Alkhanani, Atiah H. Almalki, Shafiul Haque, Saurabh Kumar Sharma, R. K. Brojen Singh, Md. Zubbair Malik

**Affiliations:** 1 Amity Institute of Neuropsychology & Neurosciences, Amity University, Noida 201303, India; lalwaniamit3711@gmail.com; 2 School of Life Sciences, Jawaharlal Nehru University, New Delhi 110067, India; krishnan.kushagra@gmail.com; 3 Department of Medical Laboratory Technology, Faculty of Applied Medical Sciences, Jazan University, Jazan 45142, Saudi Arabia; sbagabir@jazanu.edu.sa; 4 Emergency Service Department, College of Applied Sciences, AlMaarefa University, Riyadh 11597, Saudi Arabia; mkhanani@mcst.edu.sa; 5 Department of Pharmaceutical Chemistry, College of Pharmacy, Taif University, Taif 21944, Saudi Arabia; ahalmalki@tu.edu.sa; 6 Addiction and Neuroscience Research Unit, College of Pharmacy, Taif University, Taif 21944, Saudi Arabia; 7 Research and Scientific Studies Unit, College of Nursing and Allied Health Sciences, Jazan University, Jazan 45142, Saudi Arabia; shafiul.haque@hotmail.com; 8 Bursa Uludağ University Faculty of Medicine, Görükle Campus, Nilüfer, Bursa 16059, Turkey; 9 School of Computer & Systems Sciences, Jawaharlal Nehru University, New Delhi 110067, India; 10 School of Computational & Integrative Sciences, Jawaharlal Nehru University, New Delhi 110067, India; 11 Department of Biotechnology, Jamia Hamdard University, New Delhi 110024, India

**Keywords:** dementia, network medicine, GWAS, HSP90AA1, EGFR, signal propagation

## Abstract

Dementia—a syndrome affecting human cognition—is a major public health concern given to its rising prevalence worldwide. Though multiple research studies have analyzed disorders such as Alzheimer’s disease and Frontotemporal dementia using a systems biology approach, a similar approach to dementia syndrome as a whole is required. In this study, we try to find the high-impact core regulating processes and factors involved in dementia’s protein–protein interaction network. We also explore various aspects related to its stability and signal propagation. Using gene interaction databases such as STRING and GeneMANIA, a principal dementia network (PDN) consisting of 881 genes and 59,085 interactions was achieved. It was assortative in nature with hierarchical, scale-free topology enriched in various gene ontology (GO) categories and KEGG pathways, such as negative and positive regulation of apoptotic processes, macroautophagy, aging, response to drug, protein binding, etc. Using a clustering algorithm (Louvain method of modularity maximization) iteratively, we found a number of communities at different levels of hierarchy in PDN consisting of 95 “motif-localized hubs”, out of which, 7 were present at deepest level and hence were key regulators (KRs) of PDN (HSP90AA1, HSP90AB1, EGFR, FYN, JUN, CELF2 and CTNNA3). In order to explore aspects of network’s resilience, a knockout (of motif-localized hubs) experiment was carried out. It changed the network’s topology from a hierarchal scale-free topology to scale-free, where independent clusters exhibited greater control. Additionally, network experiments on interaction of druggable genome and motif-localized hubs were carried out where UBC, EGFR, APP, CTNNB1, NTRK1, FN1, HSP90AA1, MDM2, VCP, CTNNA1 and GRB2 were identified as hubs in the resultant network (RN). We finally concluded that stability and resilience of PDN highly relies on motif-localized hubs (especially those present at deeper levels), making them important therapeutic intervention candidates. HSP90AA1, involved in heat shock response (and its master regulator, i.e., HSF1), and EGFR are most important genes in pathology of dementia apart from KRs, given their presence as KRs as well as hubs in RN.

## 1. Introduction

Dementia is a syndrome associated with a deadly group of diseases claiming thousands of lives worldwide every year and contributing significantly to the global burden of disease (GBD) in a steep upward trend with aging [1]. It is manifested by a chronic or a progressive degeneration of cognitive function which affects thinking, memory, orientation, calculation, comprehension, learning capacity, judgment and language without affecting the consciousness of an individual [2,3]. Cessation in functioning of healthy neurons and destruction of their connections with the other cells present in the brain results in this syndrome [4]. Degenerative dementias have been associated with many neurological/neurodegenerative disorders (NDs), most of which are found to be proteinopathies (tauopathy, synucleinopathy, etc.), where aggregates of abnormal proteins, such as amyloid beta-peptides, α-synuclein, tau protein, etc., tend to settle in the brain with the progression of age [5,6].

Aging impacts organism proteome profiles usually by disturbing protein complexes involved in stress responses [7]. These proteinopathies become prominent with progression in age, which establishes age as a crucial factor in the development of dementias [2,3]. Alzheimer’s disease (AD), which is the most common cause of dementia (60–70% of the cases), is most prevalent in older individuals (usually above the age of 65, but some variants have been found to be originating as early as 40 years of age), whereas frontotemporal dementia (FTD) usually starts at a younger age (45–65 years), and Lewy body dementia—or dementia with Lewy bodies (DLB)—is another highly prevalent form of dementia, usually starting after the age of 50 [8,9].

Abnormal proteins causing these proteinopathies are result of a fault in their origin, i.e., genes. Various research studies implicate either direct or indirect involvement of a number of genes in dementia. APP, PS-1, PS-2 and APOE4 are four major genes found to be associated with AD, while genes such as TDP-43, FUS, MAPT, GRN, C9orf72, TMEM106B and VCP have been found to be commonly implicated in pathology of some forms of FTD; whereas an increased risk of LBD might be inherited through SNCA, GBA or APOE e4 gene otherwise, which is not usually considered a genetic disorder [5,10,11,12,13,14,15]. NOTCH3 is a defective gene implicated in pathology of cerebral autosomal dominant arteriopathy with subcortical infarcts and leukoencephalopathy (CADASIL), which is a rare form of vascular dementia (VD) [16]. And in a limited-sample sized genome wide associated study (GWAS) SH3RF3 and CSMD1 have been implicated as risk factors in the development of human immunodeficiency virus (HIV)-associated dementia [17]. Though not everything is known about mode of inheritance in dementias, most of them exhibit an autosomal dominant form [18,19,20]. Additionally, it must be kept in mind that some of the forms might not have any strong genetic origin (majority of cases of FTD-, VD- and HIV-associated dementia) and might be caused to various physiological traumas, infections or indirect genetic involvement [6,21].

Rather than working as independent entities, these genes, through a spectrum of interactions, function as an organized system, exhibiting complex characteristics; hence, it is a complex phenomenon. To simplify this complex genetic disorder (where genetic perturbations instead of a single gene give rise to the disease) we consider systems biology as a sophisticated approach using network biology tools (inspired from Leonard Euler’s graph theory) [22,23,24]. This approach has been widely used to explore the intricacies of AD (given to its large prevalence), but not much with other dementias, or to the disorders causing dementia [25,26,27,28,29]. Through this study we take an integrated approach with a focus on gene–gene or protein–protein interactions (PPI) networks to explore the existence of any centrally regulating, high-impact units and their role in driving the associations among various dementia-associated disorders. We will also derive inferences on the network’s architecture, stability and signal propagation from the network’s topology. We will also investigate the interactions among drug-associated genes and high-impact regulation units of the network in dementia.

## 2. Materials and Methods

**Figure 1:**
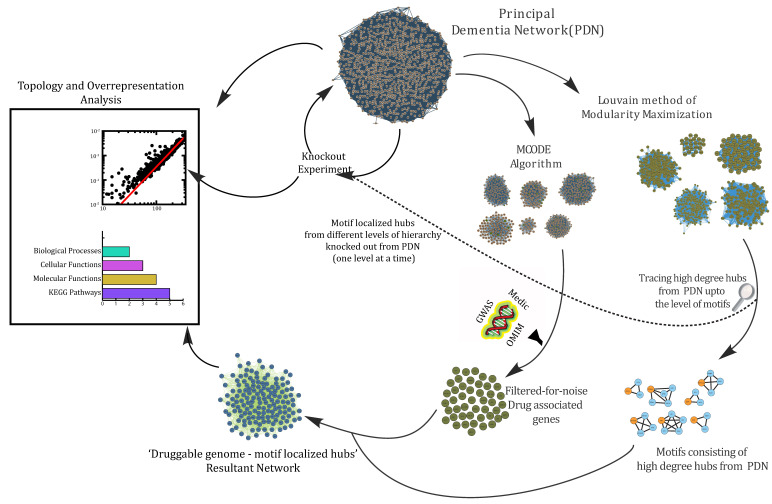
An illustrative workflow of methods and approaches carried out to study various aspects of protein–protein interactions in dementia.

Figure 1 depicts a detailed workflow of the processes carried out in this study.

### 2.1. Acquisition of Data

The Search Tool for the Retrieval of Interacting Genes/Proteins (STRING, https://string-db.org/ (accessed on 8 July 2020)) is a comprehensive database of ‘confidence scored’ protein–protein ‘functional’ interactions and gene enrichment analysis with its ‘association’ evidence in seven independent channels, meant for biochemical data, text mining, prediction estimation for interactions, co-expression data, etc. [30]. This gene database, due to its comprehensiveness, was used as a repository to extract ‘confidence-score based experimentally verified interactions’ for constructing the principal dementia network (PDN). Furthermore, we extracted genes from gene–drug interactions in different dementias using iCTNet (http://www.cs.queensu.ca/ictnet, accessed on 8 July 2020) plug in of CytoScape_3.7.2, which fetches high confidence data from the genome-wide association studies (GWAS), Online Mendelian Inheritance in Man^®^ (OMIM) and Merged Disease Vocabulary (Medic) databases [31,32,33,34].

### 2.2. Annotation Enrichment/Over-Representation Analysis

Annotation enrichment analysis helps us understand the functional biology (molecular functions, biological processes, cellular components, pathways, etc.) against the backdrop of PPIs, where the over-represented annotations associated with genes/proteins are found against the information available in various knowledge bases (GO, Reactome, KEGG, etc.) [35]. The Database for Annotation, Visualization and Integrated Discovery, DAVID_v6.8, was used in this study, which uses Fisher’s exact (modified as EASE score) as key statistical method to decipher the “biological meanings” associated with the “genes in consideration”, providing us with information on over-represented gene ontology (GO) categories against the list/set of genes fed to it (https://david.ncifcrf.gov/summary.jsp; accessed on 8 July 2020) [36]. Abundant GO categories and pathways with *p*-value ≤ 0.5 were taken as significant.

### 2.3. Construction of Protein–Protein/Gene–Gene Interaction (PPI) Networks

The aforementioned “experimentally verified interactions” were used to construct PDN in GeneMANIA_v3.5.2 plug in of CytoScape_v3.7.2. Redundant and synonymic gene names were removed from the list. Unrecognized genes in the GeneMania database were searched for synonyms in other databases such as GeneCards^®^ and UniProt.

It must be noted that STRING and GeneMANIA have been compared in various studies where they have been found having their uniqueness and merit w.r.t certain parameters, and hence the intention of using two different network construction databases is to complimentarily to picking the best features from the two [37,38,39,40]. For example, experimentally verified interactions associated with dementia were taken from STRING as it uses algorithms to grow queried network with closely related genes obtained through a combination of computed scores, based on various “literature-backed properties” with a “correction for random-interactions” making it a high-confidence repository for algorithmically tested and literature-validated interactions; meanwhile, GeneMANIA, on the other hand, with a focus on functional interactions, uses association data—such as genetic interactions, physical interactions, co-localization, co-expression and similarity of protein domains—among a queried list of genes, and hence proved useful for our “functional interactions based analysis” [30,41].

### 2.4. Characterization of Topological Properties of Networks

Topological properties help us understand the arrangement of components (nodes and edges) in a network and relevant substructures [42]. Behavior of the following topological parameters helped us in characterizing the physical properties of complex networks. These properties were obtained using the Network Analyzer and CytoNCA tools in Cytoscape_3.7.2 [43,44].

#### 2.4.1. Degree Distribution, p(k)

In a graph or a network, G =(E, V), the degree (k) of a node is defined as the number of edges connected to that node, where E and V are sets of nodes and edges, respectively. If V ={n} and $E=\left\{e_{\mathrm{ij}};i,j,i\neq j\right\}$, then the probability of degree distribution is given by the following:

(1) $$p\left(k\right)=\frac{n_{k}}{N}$$

where $n_{k}$= number of nodes with degree equal to k, and N = size of the network.

While the degree distribution of random and small-world network is a Poisson distribution (with a peak at p(k)), it deviates significantly from poisson distribution for most large networks. Degree distribution follows a power law, $p\left(k\right)\sim k^{\gamma }$, for scale-free networks, where  4≥γ≥2 and γ~2.26, indicating an inherent modular structure for hierarchical networks [42,45].

#### 2.4.2. Clustering Coefficient, c(k)

The quantification of connected triangles in a network is determined by a clustering coefficient, which helps evaluate the clustering tendency of nodes in a network (nodes in a network tend to bind their neighbors and form a number of triangular motifs, this internal connectivity and clustering therefore confers strength to the network) [46,47]. In an undirected graph, clustering coefficient for a node, i (with degree $k_{i}$), is the ratio of total number of triangular motifs formed by it with nearest neighbors to that of total number of triangular motifs in the network, as follows:

(2) $$c\left(k\right)=\frac{2m_{i}}{k_{i}\left(k_{i}-1\right)}$$

where $m_{i}$ = total number of edges for a node i among its nearest neighbors. For scale-free networks, the clustering coefficient is approximately constant, but in a hierarchical network, we can see power law against degree, that is $C\left(k\right)\sim k^{-a},\;\mathrm{with}\;a\sim 1\;$ [48].

#### 2.4.3. Neighborhood Connectivity Distribution, C_N_(k)

In a network, for a node with degree k, the neighborhood connectivity is average connectivity with its nearest neighbors [49]. $C_{N}\left(k\right)$ is mathematically represented as follows:

(3) $$C_{N}\left(k\right)=\underset{q}{\sum }\mathrm{qP}\left(q|k\right)$$

where P(q|k) = conditional probability of link belonging to node with connectivity equal to k pointing to node with connectivity equal to q. For a hierarchical network, $C_{N}\left(k\right)$ follows power law in k; $C_{N}\left(k\right)$~k^−β^ with β~0.5 and for scale-free network $C_{N}\left(k\right)$~constant [50]. Negative β could indicate disassortativity and positive β indicates assortativity [51].

#### 2.4.4. Closeness Centrality, C_C_(k)

The pace at which information is distributed from a node to the other nodes connected to it is determined by closeness centrality [52]. It indicates the proximity of a node to all other nodes in the network and is calculated as the average of the shortest path length from the node to every other node in the network. Mathematically, closeness centrality of a node m is defined as follows:

(4) $$C_{C}\left(k\right)=\frac{n}{\sum_{j}d_{\mathrm{mj}}}$$

where $d_{\mathrm{mj}}$ = geodesic path length between nodes m and j, and n = total number of nodes in the network connected to node m.

#### 2.4.5. Eigenvector Centrality, C_E_(k)

The concept of C_E_(k) relies not only on degree of node in consideration but also on degrees of the nodes it’s connected to, distinguishing highly connected neighborhoods from that of having low connections in turn emphasizing spreading power of that node in the network, thereby reducing the chance of node with high C_E_(k) from being found isolated [53]. In a network, the eigenvector centrality of a node m ($C_{E}\left(m\right)$) is proportional to the sum of (but not the average of) 

’s neighbors’ centrality [53].

(5) $$C_{E}\left(m\right)=\frac{1}{\lambda }\underset{j=\mathrm{nn}\left(m\right)}{\sum }v_{j}$$

where nn(m) designates nearest neighbors of node m in the network, λ= eigenvalue and $v_{j}$ = eigenvector. C_E_(k) score is represented by principal eigenvector of A corresponding to maximum positive eigenvalue, i.e., *λmax* [52].

#### 2.4.6. Betweenness Centrality, C_B_(k)

Betweenness centrality is directly proportional to the number of shortest/geodesic pathways passing through a node, and hence depicts the extent of the information flowing through it. It ascertains its importance in establishing the essentiality of a gene/protein much more than its degree in the network [54,55]. Nodes with high C_B_(k) are usual potential drug targets in a PPI network for a disorder and hence serves a purpose of high utility in understanding its etiology [54]. In a graph for a node, it can be determined by dividing number of shortest paths passing through that node to total number of shortest paths.

(6) $$C_{B}\left(n_{i}\right)=\underset{j<k}{\sum }g_{\mathrm{jk}}\left(n_{i}\right)/g_{\mathrm{jk}}$$

where $\;g_{\mathrm{jk}}$ designates number of shortest paths between j, and k $g_{\mathrm{jk}}\;\left(n_{i}\right)$ designates the number that node i is on.

### 2.5. MCODE (Molecular Complex Detection)-Derived Protein Complexes to Filter Drug-Actionable Genes in the Network

Aforementioned drug-associated/actionable genes were filtered for noise by tracing them in clusters obtained using stringent parameters in MCODE which is a CytoScape_3.7.2’s plugin/application, and uses an automated clustering algorithm to extract/identify densely connected regions or protein complexes in a PPI network [56]. The concept of identification of such densely connected regions in a large PPI network takes into account the differential weight of nodes, based on density of their local neighborhood, and of the local traversals arousing from a seed protein, present in that dense environment. Such clusters obtained from the main network help us in identifying the group of genes involved in particular biological processes and certain pathways.

### 2.6. Detection of Key Regulators (KRs)

Genes present PDN with degree ≥ 200 were traced down in various submodules or clusters at each hierarchical level up to the motif level, G(3, 3), using the Louvain method of modularity (Q) maximization for community detection in the “igraph” package of R [47,57,58]. Motif-associated hubs at the deepest level of hierarchy were taken as key regulators.

### 2.7. Knockout Experiment

The effects and fluctuations in organization and signal propagation in the network were evaluated by knocking out the high-degree hubs present in motifs at different hierarchical levels in the network. In a total of four eliminations, these motif-associated hubs at each hierarchical level were eliminated together, consecutively (one level at a time). Changes in topological properties of the network were then inferred using the Network Analyzer tool in Cytoscape_v3.7.2 while the eigenvector-centrality was calculated using Cytoscape’s CytoNCA plugin. This helped us understand the importance and contribution of influential hubs at different hierarchical levels towards stability and organization of the network.

### 2.8. Validation of Expression Patterns

A spatiotemporal expression heat map was generated based on the calculated expression levels of key regulators in RNAseq data from Brainspan, through BEST, a web server for brain expression spatiotemporal pattern analysis [59,60]. It will help us shed light on the progression of disease through recorded expression levels at the different life stages of a diseased individual.

## 3. Results

### 3.1. Data Acquisition and Principal Dementia Network

Using the STRING disease query for dementia (DOID: 1307), a total of 1559 disease-associated genes, having 17,262 interactions, were obtained at very high—i.e., 90%—confidence score in Cytoscape, out of which, only the aforementioned “experimentally verified genes” (STRING database experiments score ≥ 0.5 to avoid false positives) were used to create the PDN. This primary network contains gene–gene/protein–protein interactions (physical interactions = 72.96%; genetic interactions = 3.27%; pathways = 2.39%; and co-expressions = 21.37%) for dementia constructed using GeneMANIA_v3.5.2 database (data version: 13 July 2017) plug-in of Cytoscape3.7.2. It consisted of 881 nodes and 59,085 edges after duplicated edges and self-loops were removed.

### 3.2. Gene-Ontology-Based Overrepresentation Analysis

**Figure 2:**
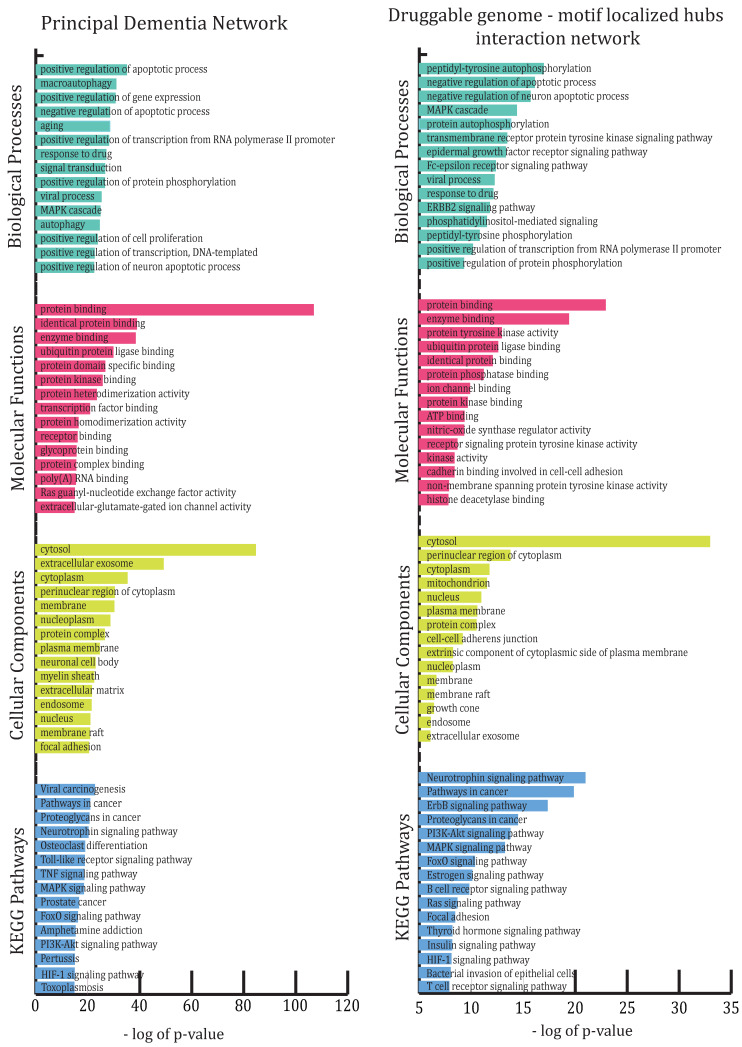
This figure illustrates the highly enriched gene ontology categories (biological processes, molecular functions, cellular components and KEGG pathways) in the principal dementia network and resultant “druggable genome–motif-localized hubs/stability” network.

All 881 genes involved in the network were subjected to enrichment analysis in DAVID according to GO-BP (biological processes), GO-MF (molecular functions), GO-CC (cellular components) and KEGG pathways (see Figure 2).

The most abundant GO-BP groups were concerned with biological regulation (positive and negative regulation of apoptotic process, positive regulation of gene expression, positive regulation of transcription from RNA polymerase II promoter, etc.), metabolic processes (aging, response to drug, response to lipopolysaccharide, inflammatory response, response to hypoxia, ephrin receptor signaling pathway, etc.) and cellular processes (macroautophagy, signal transduction, MAPK cascade, autophagy, apoptotic process, chemical synaptic transmission, cellular response to mechanical stimulus, etc.). BP categories were also found to be abundant in certain behavioral processes, such as adult locomotory behavior, behavioral fear response, social behavior, locomotory exploration behavior, etc., and few other special abundant BPs, such as circadian rhythm, angiogenesis, cerebral cortex development, neuron projection development, nervous system development, etc.

The most enriched GO-CC categories were cytosol, extracellular exosome, cytoplasm, perinuclear region of cytoplasm, membrane, nucleoplasm, protein complex, plasma membrane, neuronal cell body, etc., while most enriched GO-MF were protein binding, enzyme binding, ubiquitin protein ligase binding, protein kinase binding, protein heterodimerization activity, transcription factor binding, protein homodimerization activity, etc., among many such functions. Neurotrophin signaling pathway, osteoclast differentiation, Toll-like receptor signaling pathway, TNF signaling pathway, MAPK signaling pathway, FoxO signaling pathway and PI3K-Akt signaling pathway are some of the over-represented KEGG molecular pathways besides pathways involved in various disorders. To obtain information on all enriched categories and pathways, see Table S1–S4 in Supplementary File S1.

### 3.3. Alzheimer’s Disease and Other Dementias’ PPI Networks Exhibit Hierarchical, Scale-free Topologies

The network was analyzed for its topological properties to decipher its signal propagation and structural qualities. Power law or fractal nature was found to be exhibited by probability of degree distribution p(k)*,* clustering coefficient c(k) and neighborhood connectivity distribution C_N_(k) as a function of degree specifying the topology of the network as hierarchical and scale free. A standard statistical fitting procedure as prescribed in Clauset et al. was adopted to fit the power law on the data sets concerned with different topological parameters in the analysis [61]. Statistical *p*-values for all data sets (calculated against 2500 random samples) were larger than a critical value of 0.1 and the goodness of fit was found to be less than or equal to 0.33. While the negative values of p(k) and c(k) indicated the hierarchical nature of the network, the positive value of C_N_(k) indicated assortativity in the network, hinting at the possibility of the network being regulated through leading hubs by formation of rich clubs or their highly connected clusters. Assortativity in the network also indicates resilience in network against any removal or deletion of a hub.

(7) $$\left(\begin{array}{c} P\\ C\\ C_{n} \end{array}\right)\sim \left(\begin{array}{c} k^{-\gamma }\\ k^{-\chi }\\ k^{-\eta } \end{array}\right);\left(\begin{array}{c} \gamma_{0}\\ \chi_{0}\\ \eta_{0} \end{array}\right)\to \left(\begin{array}{c} 0.020\\ 0.211\\ 0.053 \end{array}\right)$$

Similarly, all the centralities, such as betweenness, closeness and eigenvector, were found to exhibit the fractal nature or power law as a function of degree. Their positive exponents can be said to attribute a high significance to leading hubs towards the regulation of the network.

$$\left(\begin{array}{c} C_{B}\\ C_{C}\\ C_{E} \end{array}\right)\sim \left(\begin{array}{c} k^{\beta }\\ k^{\alpha }\\ k^{\varepsilon } \end{array}\right);\left(\begin{array}{c} \beta_{0}\\ \alpha_{0}\\ \varepsilon_{0} \end{array}\right)\to \left(\begin{array}{c} 2.324\\ 0.085\\ 1.010 \end{array}\right)$$

### 3.4. Filtering Drug-Actionable Genes for Noise through Dense Clusters Obtained from the PDN

**Figure 3:**
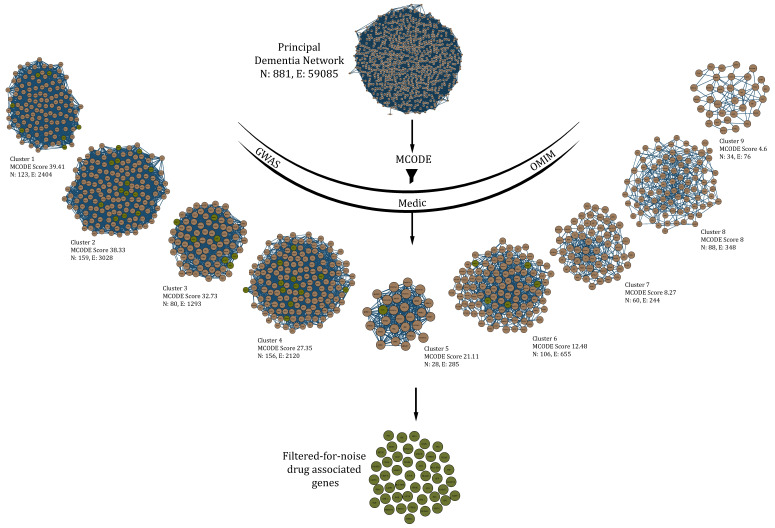
Drug-associated genes obtained from various sources (GWAS, Medic, OMIM) were “filtered for noise” in protein complexes obtained from the principal dementia network (PDN) using stringent parameters in MCODE algorithm. Only those complexes with a score greater than 10 were considered for this process.

Genes which act as drug targets can be understood as vents to introduce changes in the network. Though there are a lot of drug-actionable genes in dementia, we take specific “filtered for noise” ones only, which can be traced in dense clusters obtained through the MCODE algorithm (keeping in mind the assortative nature of the PDN) with stringent parameters (haircut = TRUE; degree cutoff = 2; node score cutoff = 0.2; K-Core = 2; maximum depth = 100). A total of 9 such clusters or protein complexes were obtained after subjecting the main network to MCODE (Figure 3).

Clusters with score ≥ 10 were used to filter all the drug-associated genes in dementia to finally obtain a total of 45 seed genes—TPI1, NGF, EIF2S1, TOMM40, IL1B, GSK3B, TNF, CD2AP, INPP5D, SNCA, CASP3, PPARG, A2M, HLA-DRA, NTRK2, CALM1, VEGFA, APBB2, IGF1R, PICALM, APP, DNMT1, ENO1, ESR1, HLA-DRB1, NTRK1, SYK, BAX, ADAM10, FERMT2, PTK2B, INSR, IGF2R, SORL1, IGF1, MAPT, MEF2C, BECN1, DPYSL2, FUS, PSEN1, BDNF, IDE, CELF1 and BIN1.

### 3.5. Key Regulators

KRs are deeply rooted hubs which serve as network’s backbone, contributing to its local and global stability. They help resist any attacks on the network by maintaining its stability, and have a key role in keeping its functional and structural integrity. Apart from their role in signal propagation and reception, KRs do serve as a medium in cross-talks between nodes even when located at a considerable distance from each other. In our modular, hierarchical, scale-free PDN, hubs by the virtue of high centrality values have an important role in controlling the flow or signal/information. At the same time, changing popularity of hubs with different activities and regulating mechanisms tells us that all leading hubs cannot act as KRs.

**Figure 4:**
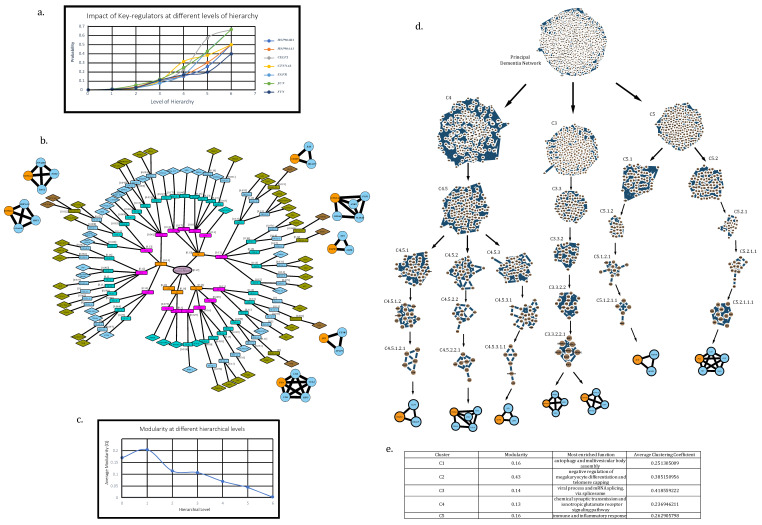
Community detection using the Louvain method of modularity (Q) maximization. (**a**) Probability graph to show the increasing impact of key regulators as we move deeper into the network’s hierarchy. (**b**) Representation of all communities obtained through the aforementioned algorithm while tracing the high-degree hubs from level 0 (principal dementia network) up to motifs at different hierarchical levels in order to find key regulators (HSP90AA1, HSP90AB1, CELF2, FYN, JUN, EGFR). Modularity values for each community have been mentioned in square bracket except for motifs (motifs have 0 modularity). (**c**) Graph showing the fall in value of modularity as we move deeper into network’s hierarchy. (**d**) Illustration of communities containing KRs at every hierarchical level in the network. (**e**) Functions and modularity of clusters at first hierarchical level.

We considered hubs with degree ≥ 200, which were traced down in modules at every level of hierarchy up to the level of motifs G (3,3) obtained after subjecting the main network to Louvain method of modularity (Q) maximization for community detection algorithm in order to find the KRs in the network. All these communities existing at different hierarchical levels leading to motifs with their modularity values (Figure 4b).

A detailed illustration displaying specific communities containing KR can be seen in Figure 4d. Average modularity values tend to decrease in value as we move deeper into the network falling, from the 1st level, Q = 0.17, to the 7th level, Q = 0.05, with the highest average at the 2nd level, Q = 0.204 (assuming PDN = 1st level) (see Figure 4c).

A total of 7 KRs were obtained in concurrence with the above definition which were HSP90AB1, HSP90AA1, CELF2, CTNNA3, EGFR, JUN and FYN. In order to obtain an estimate their impact and regulating capabilities in a module/network, we defined a probability—$P_{\mathrm{KR}}\left(x\right).$ It is the probability of a KR to have x number of edges/links in a module/network with total N number of edges/links at a given organization level, s, the equation for which can be given by the following:

(8) $$P_{\mathrm{KR}}\left(x^{\left[s\right]}\right)=\frac{x^{\left[s\right]}}{N^{\left[s\right]}};s=0,\;1,\;2,\;\ldots .m;\;m=6$$

where x^[s]^ = number of edges a KR has at a level s; N^[s]^ = total number of edges in a module/submodule or network in which that KR exists.

The calculated values of probability for KRs were found to be increasing as we move towards deeper level of hierarchical organization, hinting at an increased prominence and regulating capability of KRs as we move deeper into the network (Figure 4a).

### 3.6. Assessment of the Network’s Stability

**Figure 5:**
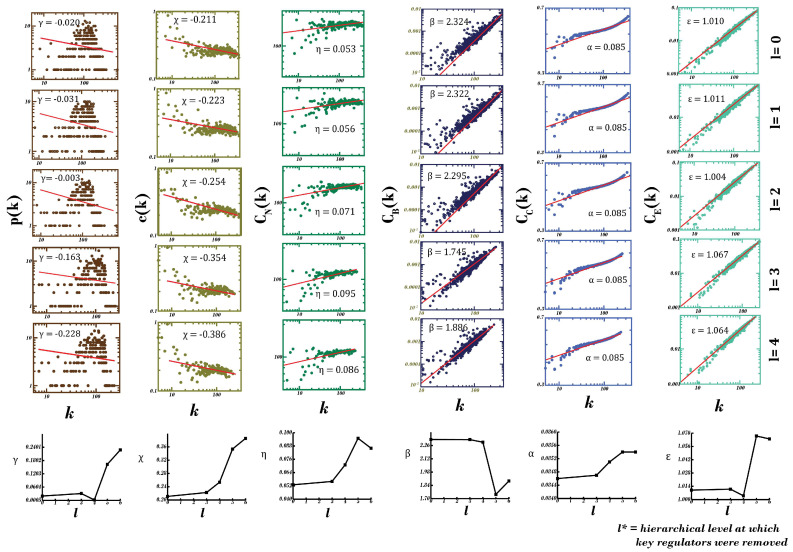
Analyses of topological properties (degree distribution p(k), average clustering coefficient c(k), neighborhood connectivity distribution C_N_(k), betweenness centrality C_B_(k), closeness centrality C_C_(k) and eigenvector centrality C_E_(k)) of the network after subsequent elimination of high-degree, motif-localized hubs found at each hierarchical level (l). Here, l = 0 represents topological properties of the principal dementia network. This knockout experiment depicts the importance of motif-localized hubs in the principal dementia network, as the network converts into a scale-free network from a hierarchical scale-free network, with subsequent eliminations showing its loss in resilience.

Knockout experiment is a technological manifestation of the traditional approach to study gene-regulatory relationships through silencing or reducing the expression levels of a particular gene, keeping all other environmental parameters constant [62]. In order to comprehend the dependence of the network’s structural stability and resilience on motif-localized hubs at different levels of hierarchical organization, we performed their knockouts (all the hubs present at a level at one time) subsequently from the PDN, starting at the 3rd level to deepest level, i.e., the 6th (considering PDN at level 0). Topological properties were studied after each elimination to infer the behavior of the resulting network in absence of, or silencing, the desired genes (see Figure 5).

$$\left(\begin{array}{c} \gamma_{i}\\ \chi_{i}\\ \eta_{i} \end{array}\right)\to \left(\begin{array}{c} 0.020-0.228\\ 0.211-0.386\\ 0.053-0.086 \end{array}\right);\left(\begin{array}{c} \beta_{i}\\ \alpha_{i}\\ \varepsilon_{i} \end{array}\right)\to \left(\begin{array}{c} 2.324-1.886\\ 0.085-0.085\\ 1.010-1.064 \end{array}\right);\;i=0,\;1,\;2,\;3,\;4\;\left(5\right)$$

A rise in the value and change in sign (from negative to positive) of γ (exponent of degree distribution) was observed (0.020–0.228), with subsequent eliminations indicating a higher significance of motif-localized hubs in the network and a shift from a hierarchical scale-free nature to a scale-free nature, indicating a loss of resilience [63]. Furthermore, an increase in the exponent of the clustering coefficient, i.e., χ (0.211–0.386), and in that of neighborhood connectivity, i.e., η (0.053–0.095, till 5th hierarchical level), hints at increasing compactness of the network with modules gaining in regulating ability compared with existing hubs with each subsequent elimination. This increase in assortativity can be explained as low-degree nodes—earlier associated with high-degree hubs—have started gaining in connections, giving rise to modules. A little fall in assortativity η (0.095–0.086) after last elimination (consisting of KRs) indicates loss of connections of low-degree nodes establishing a higher importance of KR genes and a further jolt to whatever little resilience left in the network.

A continuous fall in exponent of betweenness centrality, i.e., β (2.324–1.886), after each elimination hints at importance of eliminated hubs or decreased control of existing hubs towards regulation of the network [64]. Values of exponents of closeness (α: 0.08464–0.08545) and eigenvector (ε: 1.01028–1.06447) centralities kept on increasing (with very little extent) after each subsequent elimination, indicating a slightly faster propagation of signal attributed to increase in α (helping compensate for lost organization and properties due to knockout) and formation of stronger links in the network reducing chances of any node being found isolated, attributed to increase in value of ε [52,53]. This experiment establishes the high importance of motif-localized hubs in maintaining network’s stability and hierarchical, scale-free nature, as their knockout brings the network to the verge of breakdown, where modules gain prominence in its regulation over existing hubs—ultimately causing harm to the network’s architecture and signal propagation.

### 3.7. Interaction Analysis of Druggable Genome and Network’s Stability

**Figure 6:**
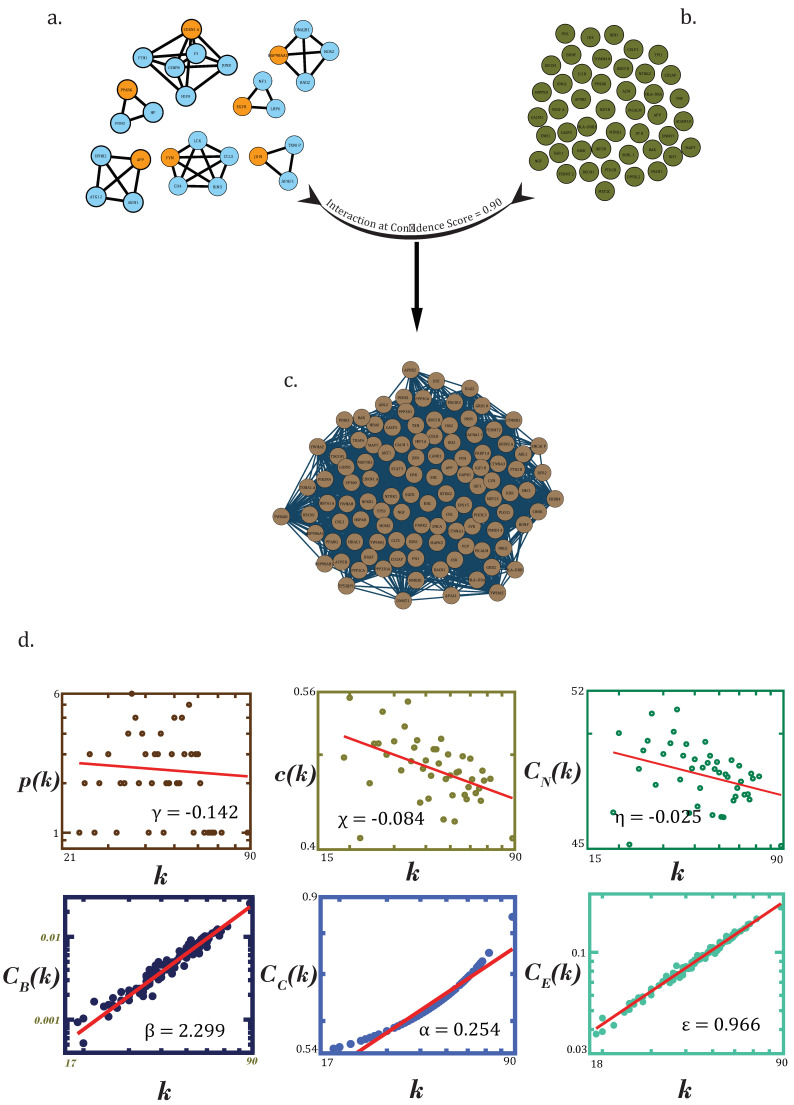
(**a**) All motif-localized hubs (total of 95) in the principal dementia network. (**b**) Noise-filtered drug-associated genes. (**c**) Resultant network (**a**–**c**). Construction of resultant “druggable genome–motif-localized hubs/stability” network consisting of high confidence, and interactions among motif-localized hubs were experimentally verified and filtered for noise drug-associated genes. (**d**) Topological properties (degree distribution p(k), average clustering coefficient c(k), neighbourhood connectivity distribution C_N_(k), betweenness centrality C_B_(k), closeness centrality C_C_(k) and eigenvector centrality C_E_(k) of resultant network.

Through “drug-associated genes”, changes can be introduced in the network—essentially making them “key-drivers”, determining the fate of the disease. Through interaction of these drug-associated genes (filtered for noise) (Figure 6b) with KRs and genes present in motifs at last level of hierarchy (representing key regulators of network’s stability and architecture) (Figure 6a), we tried to find the mediators or facilitators of high-impact interactions in the network. These genes were checked for connectivity at very high confidence score (95%) in the STRING database. Genes involved in “experimentally verified interactions” (stringdb experiments score ≥ 0.5 to avoid false positives) were taken as seed-genes for network to be grown in GeneMANIA_v3.5.2 plugin of CytoScape. This resultant network (RN) consisted of 109 genes with a total of 2433 interactions (Figure 6c), made up of physical (66.37%), genetic (2.70%), pathways (5.11%) and co-expression (25.82%) interactions.

Using the earlier used standard statistical fitting procedure, power law or fractal nature was found to be exhibited by probability of degree distribution p(k)*,* clustering coefficient c(k) and neighborhood connectivity distribution C_N_(k) as a function of degree with their negative exponents indicating the hierarchical scale-free nature of the network, exhibiting disassortativity hinting at no probability of formation of rich clubs or highly connected clusters (Figure 6d). This network would rather be regulated by high-degree hubs than any rich clubs or clusters.

(9) $$\left(\begin{array}{c} P\\ C\\ C_{n} \end{array}\right)\sim \left(\begin{array}{c} k^{-\gamma }\\ k^{-\chi }\\ k^{-\eta } \end{array}\right);\left(\begin{array}{c} \gamma_{0}\\ \chi_{0}\\ \eta_{0} \end{array}\right)\to \left(\begin{array}{c} 0.142\\ 0.084\\ 0.025 \end{array}\right)$$

All the centrality measures were found to exhibit the fractal nature as a function of degree. Positive exponents of these centrality measures can be said to attribute a high significance to leading hubs towards regulation of the RN. UBC, EGFR, APP, CTNNB1, NTRK1, FN1, HSP90AA1, MDM2, VCP, CTNNA1 and GRB2 were the top genes selected on the basis of degree and C_B_(k) (≥0.010) (rounded to 3 decimal places) (Table 1).

(10) $$\left(\begin{array}{c} C_{B}\\ C_{C}\\ C_{E} \end{array}\right)\sim \left(\begin{array}{c} k^{\beta }\\ k^{\alpha }\\ k^{\varepsilon } \end{array}\right);\left(\begin{array}{c} \beta_{0}\\ \alpha_{0}\\ \varepsilon_{0} \end{array}\right)\to \left(\begin{array}{c} 2.299\\ 0.254\\ 0.966 \end{array}\right)$$

In RN, peptidyl–tyrosine autophosphorylation, negative regulation of apoptotic and neuron apoptotic process, MAPK cascade, protein and enzyme binding, protein tyrosine kinase activity, ubiquitin protein ligase binding, cytosol, perinuclear region of cytoplasm, cytoplasm and mitochondrion were some of the over-represented GO categories; meanwhile, the neurotrophin signaling pathway, pathways in cancer and ErbB signaling pathway were some of the over-represented KEGG pathways. Information on other enriched categories can be found in Table S1–S4 in Supplementary File S2.

### 3.8. Validation of Key Regulators’ Expression Patterns

**Figure 7:**
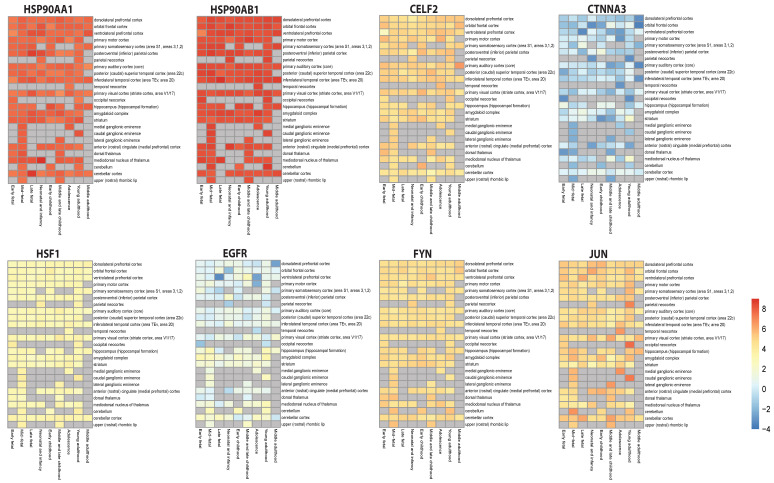
Spatiotemporal expression heatmap of key regulators from BEST tool.

The BEST tool [60] was used to investigate the expression pattern of KRs and master modulator of heat shock response (HSR), i.e., HSF1 as shown in Figure 7. The heat map plots showing their expression distribution in brain are shown in Figure 7.

Results show high and continuous expression of heat shock proteins (HSP90AA1 and HSP90AB1) in most of the regions of the brain throughout life, especially in the cortex region, while HSF1 shows a slightly higher expression than normal. EGFR showed suppressed expression in some parts of the brain and slightly higher expression in other parts, while CTNNA3 shows highly suppressed and fluctuating expression throughout life. CELF2, FYN and JUN displayed higher expressions, with a lot of fluctuations in JUN’s expression levels in almost all the brain’s parts, with higher expression in the parietal, temporal and occipital neocortex, and in ganglionic eminences.

## 4. Discussion

Dementia is one of the “not so silent” epidemics, claiming thousands of lives worldwide. This syndrome is associated with several complex NDs, which are caused by complex genetic interactions. Our investigation into the interactions and topology of PDN revealed its hierarchical, scale-free and assortative nature as probability of degree distribution p(k), clustering coefficient c(k) and neighborhood connectivity distribution C_N_(k), exhibiting a fractal nature as a function of degree. The network’s centrality measures, i.e., betweenness centrality C_B_(k), closeness centrality C_C_(k) and eigenvector centrality C_E_(k), also exhibited fractal nature as a function of degree, with positive exponents reflecting the importance of highly connected nodes (hubs) towards network’s regulation. Positive regulation of gene expression and apoptotic process, negative regulation of apoptotic process, macroautophagy, aging, protein binding, enzyme binding, identical protein binding, ubiquitin protein ligase binding, cytosol, extracellular exosome, cytoplasm and perinuclear region of cytoplasm were some of the over-represented GO categories in the network; meanwhile, pathways in cancer, the neurotrophin signaling pathway and osteoclast differentiation were some of the over-represented KEGG pathways.

Complex networks at their core have these recurrent basic geometrical units encapsulating their evolutionary principles—”motifs”—which are “patterns for which the probability P of appearing in a randomized network an equal or greater number of times than in the real network is lower than a cutoff value” [65,66]. Significance of these structures tended to increase with the increase in the size of the network; so, keeping in mind the vastness and complexity of our PDN, it becomes imperative to investigate the involved motifs and decipher their influence on system-wide dynamics [65]. Combining this fact with “regulating influence” of hubs, we traced a total of 109 high-degree hubs (degree ≥ 200) up to the level of motifs G (3, 3) using the Louvain method of modularity (Q) maximization algorithm for community detection, as this clustering algorithm is more suitable for large complex networks compared with other common community detection algorithms.

A total of 7 levels of hierarchy in the network (considering PDN at 1st level) were obtained, with average modularity value (Q) falling from the 1st to the 7th level (except in some cases), where hubs present in motifs at the deepest levels were taken as key regulators (CELF2, CTNNA3, JUN, HSP90AB1, HSP90AA1, EGFR and FYN). KRs’ regulating influence increased as we went deeper into the network. Motif-localized hubs at different levels of hierarchy do have a prominent role in the network’s regulation and stability as their knockout brought about a change in the network’s nature from “hierarchical scale-free” to “scale-free” and gave an upper hand to modules rather than existing hubs towards network’s regulation.

Druggable genome introduces external changes into the network, and to understand their influence over the network’s stability, we studied high-confidence interactions among drug-associated genes, and earlier obtained motif-localized hubs to deduce the major checkpoints of traffic/signals in such a setting. RN formed as a result of such interactions exhibiting a hierarchical, scale-free nature with disassortativity and positive exponents for centrality measures. Peptidyl–tyrosine autophosphorylation, negative regulation of neuron apoptotic process, protein binding, enzyme binding, cytoplasm, cytosol, mitochondrion and nucleus were some of the over-represented GO categories, while pathways in cancer and neurotrophin signaling pathways were some of the over-represented KEGG pathways. Using degree and C_B_(k) as measures, we found that UBC, EGFR, APP, CTNNB1, NTRK1, FN1, HSP90AA1, MDM2, VCP, CTNNA1 and GRB2 are major hubs of RN, out of which, EGFR and HSP90AA1 can be considered as the most important genes of this study, as they not only host the major traffic of aforementioned interactions but also are KRs of PDN. Table 2 consists of formation on topology of genes which are major hubs in PDN but are also present in motifs at deepest level of hierarchy.

HSP90AA1 is one of the many heat shock proteins (as they are involved in one of the major stress responses called heat shock response (HSR)) and is a member of the HSP90 group with 3 distinct domains (each containing post translation modification sites), responsible for nucleotide binding, dimerization, and client recognition and ATP binding [67]. It is an inducible molecular chaperone with HSP90AB1 as a subsequently expressing isoform [68]. They aid other proteins with their proper folding through conformational changes in later stages to provide them with activity and stability [68]. Under normal or stressed conditions these chaperons in coordination with co-chaperons avoid protein aggregation and misfolding, while chaperons on their own majorly regulate PPI and stress-induced response by degrading aggregated or misfolded proteins (they also unfold proteins) [69,70]. HSP90, as compared with other chaperons, is rather specific, and only attaches to some specific sets of proteins [71]. A number of studies implicate HSP90 by the virtue of HSR in pathologies of AD, FTD and LBD. We found their (HSP90AA1 and HSP90AB1) high and continuous expressions in almost all parts of brain throughout the life of an individual with NDs.

AD and FTD can be collectively studied as tauopathies, as they share some common characteristics in their pathologies, one of which is presence of tau [72]. Hyperphosphorylation of tau (which causes AD) is a result of abnormal activation of GSK3 and other kinases, which most likely happens due to initial Aβ toxicity or soluble Aβ oligomers (toxic to synapses), and has been reported to induce neuronal apoptosis, mediated by p38^MAPK^ and c-JUN amino-terminal kinases or JNKs [73,74,75,76,77,78,79,80]. HSP90 has a prominent role here, as its inhibition can help achieve reduction in this abnormal phosphorylation of tau [81]. In AD, synaptic markers and their density was found to be improved when HSP90 inhibitors were used in an in vivo mouse model (improvements with respect to LTP and memory loss were also observed) [82,83]. Luo, Wenjie et al. in 2008 reported important role of HSP90 in AD as it provides functional stability to its progression through a buffering mechanism similar to that in cancer [84]. Meanwhile, in the case of FTD, mutations on chromosome 17 in human tau isoforms lead to one of its forms, i.e., FTDP-17 (FTD and Parkinsonism related to chromosome-17), also ubiquitinated and hyperphosphorylated; TDP-43 have been found to form toxic pathological aggregates, and as TDP-43 co-immunoprecipitates with HSP70 and HSP90, knockdown of these heat shock proteins can lead to its hyperphosphorylation with an increase in its C-terminal [85,86,87,88,89,90].

HSP90 has also been implicated in NDs with characteristically increased abnormal levels of α-synuclein, i.e., synucleinopathies (LBD, AD and multiple system atrophy) [91,92,93]. It has been found present in Parkinson’s disease (PD) patient’s Lewy bodies, where it colocalizes with amyloid filaments and soluble α-synuclein and promotes ATP-dependent restriction of α-syn binding to vesicles and fibril formation [94,95]. In vitro research indicates that HSP90 (in absence of ATP)—instead of forming fibrils—promotes α-syn oligomers, and these soluble, intermediate oligomers are responsible for toxicity and pathogenicity [95]. Even with all this knowledge, apart from HSP90′s scavenging role, its direct involvement in PD and LBD is not clear. Additionally, no studies implicate the role of HSP90 in VD or vascular cognitive impairment, but a single study implicates the role of HSP70 in inflammation in patients with vascular mild cognitive impairment [96].

Though Heat shock factor 1 (HSF1)—which is called the master regulator (transcriptional) of HSR has kept a very low profile throughout different levels of hierarchy (low degree and C_B_(k)), but was found present in motif with one of the KRs i.e., HSP90AB1, and hence can be said to play a very important role in network’s regulation [97,98,99]. Slightly higher and continuous expressions of HSF1 were found in individuals with NDs throughout their lives.

HSF1 is activated (by inhibition of HSP90) and mitigates stress (aging, heat, change in osmosis, etc.) by controlling HSR as it binds to upstream sequences of promoters of heat shock genes resulting in the synthesis of cytoprotective proteins, mitigating toxicity caused by abnormal proteins [100,101,102,103]. Under stress, HSF1 translocates to the nucleus from cytosol, and through changes in post-translational modifications (acetylation, phosphorylation and sumoylation) and PPI, it modulates DNA-binding transactivation, which regulates HSR [104,105,106,107]. It also upregulates the synthesis of APP, possibly due to its pro-synaptogenic nature [108]. With the progression of age, functioning of HSR is affected mainly due to defective HSF1 activation [109].

It must be noted that HSP90 and HSP70 together play an important roles in HSR as they can shut down HSF1 in absence of stressor (negative regulation) and also are important components of chaperone-mediated autophagy (CMA) machinery, which plays an important role in pathologies of neurodegenerative diseases, such as PD, Huntington’s disease and tauopathies, by preventing α-synuclein, polyQ-huntingtin and tau from accumulating [110,111,112,113,114]. Additionally, this explains higher expression levels of HSP90AA1 and HSP90AB1 against slightly increased expressions of HSF1 in people with NDs throughout their lives.

Epidermal growth factor receptor (EGFR) is another high-impact gene in our study, which shows differential and fluctuated expression in various parts of brains of people with NDs. Through various routes, it might be involved in age-related neurodegeneration, and has been implicated for its role in memory loss due to Aβ oligomer-induced activation [115,116]. Long-term synaptic plasticity is regulated by the downstream signaling pathways of EGFR, such as PI3K, Ras, etc., which are known to be disrupted by these Aβ oligomers [117,118,119]. Regarding EGFR’s relation with AD, research demonstrates the controlling ability of Aβ and presenilin (product of risk genes causing AD) over EGFR’s metabolism, as well as expression, but there is still no evidence of a direct relationship between the EGFR pathway and AD [120,121,122]. EGFR can be involved in the pathology of LBD, as it is possibly involved in synthesis of dopaminergic neurons, as it is regulated by the dopamine–EGFR signaling loop [123]. Though the literature regarding the implication of EGFR in FTD, LBD, VD and HIV-associated dementia could not be found, it has an important role to play in aging-related metabolism. Through involvement in insulin/IGF-1/GH signaling systems, energy metabolism, hepatic function (hepatocyte proliferation, etc.) and other important processes, EGFR regulates aging-related metabolism [115].

Fluctuating ‘expression levels’ of other KRs—such as CTNNA3 and JUN—in various brain parts in of people with NDs hints at nature of their contribution in progression of dementia.

## 5. Conclusions

This study depicts the prominence of motif-localized hubs existing at different levels of hierarchy towards a hierarchical, scale-free principal dementia network’s stability and resilience. Seven such hubs (HSP90AA1, HSP90AB1, CELF2, CTNNA3, JUN, EGFR, FYN), existing at the deepest level of hierarchies, are taken as key regulators of PDN—due to their high influence over it. Through a comprehensive methodology and review of the literature, we demonstrated how the HSP90 genes/proteins, which are involved in the heat shock response, regulated by HSF1 and EGFR gene/proteins, do claim a major authority over regulation and signal propagation in the PDN, along with over interactions among druggable genomes in dementia and in motif-localized hubs (the network’s stability). Differential expression levels of these KRs in various brain parts of people with NDs through different stages of their lives provide us with valuable insights on their contribution in the progression of neurodegenerative dementias. We also delved into various biological processes associated with PDN through overrepresentation analysis using GO annotation tools and a review of the literature, which tells about its involvement in apoptotic processes, kinase activity, behavioral processes and dementia’s association with aging metabolism, among many others. This study can serve as a quality stimulus for in vitro and in vivo studies of dementia.

## Figures and Tables

**Table 1:** High-degree hubs with topological properties from resultant “druggable genome–motif-localized hubs” network.

Gene	Name	Gene Ontology Annotation	Degree (k)	c(k)	C_N_(k)	C_B_(k)	C_C_(k)	C_E_(k)
UBC	ubiquitin C	protease binding	88	0.419801	45.125	0.025	0.84375	0.171251
EGFR	epidermal growth factor receptor	identical protein binding and protein kinase activity	72	0.475352	48.08333	0.013	0.75	0.148904
APP	amyloid beta precursor protein	identical protein binding and enzyme binding	66	0.454079	47.07576	0.012	0.72	0.133501
CTNNB1	catenin beta 1	DNA-binding transcription factor activity and binding	68	0.473661	47.98529	0.012	0.72973	0.140163
NTRK1	neurotrophic receptor tyrosine kinase 1	protein homodimerization activity and protein kinase activity	65	0.465865	47.30769	0.012	0.715232	0.132686
FN1	fibronectin 1	heparin binding and protease binding	63	0.461342	47.28571	0.011	0.705882	0.128571
HSP90AA1	heat shock protein 90kDa alpha family class A member 1	identical protein binding	67	0.483492	48.20896	0.010	0.724832	0.139365
MDM2	MDM2 proto-oncogene	identical protein binding and ligase activity	59	0.456458	46.86441	0.010	0.687898	0.119216
VCP	valosin-containing protein	signaling receptor binding	61	0.477049	47.95082	0.010	0.696774	0.125994
CTNNA1	catenin alpha 1	actin filament binding	58	0.455535	47.2069	0.010	0.683544	0.117217
GRB2	growth factor receptor-bound protein 2	protein kinase binding	61	0.472678	47.77049	0.010	0.696774	0.125459

**Table 2:** Key regulators and genes present with them in motifs at 6th level with topological properties from principal dementia network.

Gene	Name	Gene Ontology Annotation	Degree (k)	c(k)	C_N_(k)	C_B_(k)	C_C_(k)	C_E_(k)
ANK2	ankyrin 2, neuronal	protein kinase binding and structural constituent of cytoskeleton	153	0.23787	164.797	9.84 × 10^−4^	0.54624	0.03467
APAF1	apoptotic peptidase activating factor 1	identical protein binding and ADP binding	131	0.23864	163.863	7.37 × 10^−4^	0.53922	0.02975
BAG2	BCL2 associated athanogene 2	identical protein binding and chaperone binding	137	0.25537	165.263	8.11 × 10^−4^	0.54121	0.03184
CCL5	C-C motif chemokine ligand 5	protein homodimerization activity and chemokine activity	126	0.36648	158.738	4.94 × 10^−4^	0.53528	0.0271
CD4	CD4 molecule	protein homodimerization activity and enzyme binding	148	0.25896	162.304	0.00104	0.54321	0.03311
**CELF2**	CUGBP, Elav-like family member 2	nucleic acid binding and RNA binding	278	0.23663	163.838	0.00327	0.59259	0.06322
**CTNNA3**	catenin alpha 3	structural molecule activity and beta–catenin binding	205	0.24017	161.654	0.0022	0.56374	0.04577
DNAJB1	DnaJ heat shock protein family (Hsp40) member B1	unfolded protein binding and ATPase binding	125	0.25639	163.832	6.87 × 10^−4^	0.5379	0.02853
**EGFR**	epidermal growth factor receptor	identical protein binding and protein kinase activity	316	0.23984	165.997	0.004	0.609	0.07338
FGF1	fibroblast growth factor 1	growth factor activity and Hsp70 protein binding	153	0.2254	158.529	0.00113	0.54591	0.03315
**FYN**	FYN proto-oncogene, Src family tyrosine kinase	transferase activity, transferring phosphorus-containing groups and protein tyrosine kinase activity	231	0.26934	172.139	0.00204	0.57516	0.05493
HDAC9	histone deacetylase 9	transcription factor binding and histone deacetylase binding	122	0.27476	176.23	5.79 × 10^−4^	0.53528	0.02986
HSF1	heat shock transcription factor 1	DNA-binding transcription factor activity and chromatin binding	92	0.3022	166.761	2.95 × 10^−4^	0.52569	0.02156
**HSP90AA1**	heat shock protein 90kDa alpha family class A member 1	identical protein binding	293	0.27411	172.635	0.00305	0.59823	0.07174
**HSP90AB1**	heat shock protein 90kDa alpha family class B member 1	protein kinase binding	207	0.30772	176.865	0.0015	0.56519	0.05223
HSPA1A	heat shock protein family A (Hsp70) member 1A	ubiquitin protein ligase binding	108	0.35722	193.324	3.41 × 10^−4^	0.53108	0.02952
IL34	interleukin 34	cytokine activity and macrophage colony-stimulating factor receptor binding	32	0.22379	160.719	4.95 × 10^−5^	0.50372	0.00696
**JUN**	Jun proto-oncogene, AP-1 transcription factor subunit	sequence-specific DNA binding	298	0.23117	161.597	0.00383	0.59986	0.06713
LCK	LCK proto-oncogene, Src family tyrosine kinase	identical protein binding and protein kinase activity	150	0.30318	174.307	7.34 × 10^−4^	0.54422	0.03627
LRP6	LDL receptor related protein 6	protein homodimerization activity and signaling receptor binding	98	0.23312	159.214	7.21 × 10^−4^	0.52695	0.02125
NF1	neurofibromin 1	binding and phosphatidylcholine binding	119	0.2286	162.832	6.87 × 10^−4^	0.53495	0.02656
NOS2	nitric oxide synthase 2	protein homodimerization activity and oxidoreductase activity	107	0.23911	165.486	6.23 × 10^−4^	0.53012	0.02444
RIN3	Ras and Rab interactor 3	GTPase activator activity and Rab guanyl–nucleotide exchange factor activity	43	0.29236	158.86	7.03 × 10^−5^	0.50286	0.00939
RPS6KB2	ribosomal protein S6 kinase B2	transferase activity, transferring phosphorus-containing groups and protein tyrosine kinase activity	112	0.27622	163.482	4.95 × 10^−4^	0.52916	0.02593
TXNIP	thioredoxin interacting protein	ubiquitin protein ligase binding and enzyme inhibitor activity	166	0.25936	171.554	9.43 × 10^−4^	0.55103	0.03948
UBE4A	ubiquitination factor E4A	ligase activity and ubiquitin–ubiquitin ligase activity	188	0.23603	158.957	0.00151	0.55802	0.04172
VLDLR	very low-density lipoprotein receptor	calcium ion binding	139	0.23762	162.698	8.79 × 10^−4^	0.54087	0.03118

## Data Availability

Not applicable.

## Supplementary Materials

The following supporting information can be downloaded at: https://www.mdpi.com/article/10.3390/biom12030451/s1, Supplementary File S1 (Table S1: Biological Processes; Table S2: Molecular Functions; Table S3: Cellular Components; Table S4: KEGG pathways); Supplementary File S2 (Table S1: Biological Processes; Table S2: Molecular Functions; Table S3: Cellular Components; Table S4: KEGG pathways).
